# Are enterococcal bloodstream infections an independent risk factor for a poorer 5-year survival or just a marker for severity of illness?—The Munich multicentric enterococci cohort

**DOI:** 10.1128/spectrum.02585-23

**Published:** 2023-10-04

**Authors:** Kathrin Rothe, Tobias Bachfischer, Siranush Karapetyan, Alexander Hapfelmeier, Milena Wurst, Sabine Gleich, Karl Dichtl, Roland M. Schmid, Julian Triebelhorn, Laura Wagner, Johanna Erber, Florian Voit, Rainer Burgkart, Andreas Obermeier, Ulrich Seibold, Dirk H. Busch, Patrick C. Rämer, Christoph D. Spinner, Jochen Schneider

**Affiliations:** 1 Institute for Medical Microbiology, Immunology and Hygiene, University Hospital rechts der Isar, Technical University of Munich, School of Medicine, Munich, Germany; 2 Department of Internal Medicine II, University Hospital rechts der Isar, Technical University of Munich, School of Medicine, Munich, Germany; 3 Institute of General Practice and Health Services Research, University Hospital rechts der Isar, School of Medicine, Technical University of Munich, Munich, Germany; 4 Institute of AI and Informatics in Medicine, University Hospital rechts der Isar, School of Medicine, Technical University of Munich, Munich, Germany; 5 Public Health Service, City of Munich, Munich, Germany; 6 Max von Pettenkofer-Institut für Hygiene und Medizinische Mikrobiologie, Medizinische Fakultät, Ludwig-Maximilians-Universität München, Munich, Germany; 7 Diagnostic and Research Institute of Hygiene, Microbiology and Environmental Medicine, Medical University of Graz, Graz, Austria; 8 German Centre for Infection Research (DZIF), partner site Munich, Munich, Germany; 9 Clinic of Orthopaedics and Sports Orthopaedics, University Hospital rechts der Isar, Technical University of Munich, School of Medicine, Munich, Germany; 10 Division of Infectious Diseases, Department of Medicine IV, Hospital of the LMU Munich, Munich, Germany; 11 Department of Hospital Hygiene and Infection Control, Munich Municipal Hospital Group, Munich, Germany; University Paris-Saclay, AP-HP Hôpital Antoine Béclère, Service de Microbiologie, Institute for Integrative Biology of the Cell (I2BC), CEA, CNRS, Clamart, France

**Keywords:** enteroccocal bloodstream infections, 5-year survival, *Enterococcus faecium*, vancomycin resistant *Entercoccus faecium*, disease severity

## Abstract

To assess the long-term survival of patients with enterococcal bloodstream infections (BSI), encompassing various species and resistance patterns compared to *Escherichia coli* (*E. coli*) BSI. Between 2010–2019, 3,290 enterococcal and 3,415 *E. coli* BSI were retrospectively screened in seven hospitals in Munich, Germany. All vancomycin-resistant (VRE), vancomycin/linezolid-resistant (LVRE), and linezolid-resistant (LRE) *Enterococcus faecium* (ECFM) BSI were included. *Enterococcus faecalis* (ECFA), vancomycin/linezolid-susceptible ECFM, and *E. coli* BSI were randomly assigned. Cox regression analysis was used to assess survival as the primary endpoint and was adjusted for limiting prognostic factors, which were measured for their importance using a random forest model (RFM). We analyzed 952 patients with 916 episodes of enterococcal BSI and 193 episodes of *E. coli* BSI. RFM identified multimorbidity and markers for disease severity as most indicative of low survival in enterococcal BSI. The 5-year survival was significantly lower for enterococcal BSI than for *E. coli* BSI (23.9% vs 42.3%; *P* < 0.001). This difference remained significant in the Cox regression analysis after adjusting for 17 prognostic factors and excluding patients with limited life expectancy (metastatic tumor disease, Charlson-Comorbidity-Index ≥5). Adjusted 5-year survival was similar for *E. coli* and ECFA but significantly different between ECFA and ECFM (29.2% vs 21.7%; *P* = 0.002). The analysis conducted on monomicrobial ECFM and VRE BSI indicated that their respective 5-year survival was similar (19.6% vs. 21.2%; *P* = 0.753). ECFM BSI seems to be an independent risk factor for poor long-term survival. However, additional vancomycin resistance does not appear to be a contributing factor.

**IMPORTANCE:**

The present study provides a substantial contribution to literature, showing that patients with enterococcal bloodstream infections (BSI) have a lower survival rate than those with *Escherichia coli* (*E. coli*) bloodstream infections after adjusting for 17 limiting prognostic factors and excluding patients with a limited life expectancy [metastatic tumor disease, Charlson Comorbidity Index (CCI) (greater than or equal to) 5]. This difference in the 5-year long-term survival was mainly driven by *Enterococcus faecium* (ECFM) bloodstream infections, with vancomycin resistance not being a significant contributing factor. Our findings imply that *E. faecium* bloodstream infections seem to be an independent risk factor for poor long-term outcomes. As such, future research should confirm this relationship and prioritize investigating its causality through prospective studies.

## INTRODUCTION

Mortality due to bloodstream infections (BSI) is high, with a 30-day mortality rate of approximately 15%, which is expected to triple over a period of 3 years (1). As a core member of the normal flora of the gastrointestinal tract, *Enterococcus* species represent the third leading cause of nosocomial BSI in the United States (2). Most enterococcal BSIs arise following urinary tract infections, intraabdominal infections, device infections, and endocarditis (3). The mean 30-day mortality rate for enterococcal BSI is high, at approximately 25%, according to literature (4 – 9), and appears to vary significantly depending on the underlying *Enterococcus* species and resistance phenotype involved. Studies (9, 10) indicate that the 30-day mortality rate is the highest for vancomycin-resistant (VRE) *Enterococcus faecium* (ECFM), with rates of up to 57%, followed by vancomycin-susceptible *E. faecium* and *Enterococcus faecalis* (ECFA), with rates of 34% and 21%, respectively. Recently, there has been an increased burden of BSI caused by multidrug-resistant *Enterococcus* species, such as VRE, linezolid-resistant (LRE) *E. faecium*, and vancomycin/linezolid-resistant (LVRE) *Enterococcus faecium* (11 – 13). It is still debatable whether an increase in multidrug resistance in enterococcal BSI is associated with higher attributable mortality (14 – 16). It was suggested that this is because patients with VRE or LVRE BSI are usually severely ill and have multiple comorbidities, which can significantly confound the assessment of mortality risk (17). This study aimed to evaluate the long-term survival after enterococcal BSIs in relation to the causative species, resistance patterns, severity of illness, and comorbidities of the affected patients. To compare our findings of enterococcal BSI with those of other common pathogens that cause BSI, we used episodes of BSI with *Escherichia coli* (*E. coli*) as a control group, since *E. coli* is a common pathogen of BSI that also spreads from the gastrointestinal tract and causes similar range of infections as the *Enterococcus* species.

## RESULTS

### Baseline characteristics

In total, 916 episodes of enterococcal BSI and 193 episodes of non-recurrent *E. coli* BSI [with a proportion of 19.2% (37/193) extended-spectrum beta-lactamase (ESBL) in the *E. coli* BSI group] were included. Of the 916 enterococcal episodes, 529 were complicated enterococcal BSI, including 11 LVRE, 193 VRE, and 33 LRE episodes; 56 were two different enterococcal species within one episode; and 236 were recurrent enterococcal episodes. The remaining 387 episodes of enterococcal BSI were classified as uncomplicated, with 194 and 193 caused by ECFM and ECFA, respectively. Hemodialysis for chronic kidney disease (*P* = 0.007), stem cell transplantation (SCT) (*P* < 0.001), and metastatic malignancy (*P* = 0.002) were more common in patients with enterococcal BSI compared to those with *E. coli* BSI. Polymicrobial bacteremia occurred in 407/916 (44.4%) of enterococcal BSI and 38/193 (19.7%) of *E. coli* BSI. The high rate of polymicrobial BSI might result from the high proportion of infectious foci in the GI tract (Enterococci 21.5%, *E. coli* 12.4%) and from vascular catheters (Enterococci 19.1%, *E. coli* 14.5%). In such cases, polymicrobial BSI is not uncommon. This arises from the composition of our patient collective of over two-thirds ICU and hemato-oncology patients. All baseline characteristics, including details on *E. coli and* enterococcal BSI (i.e., underlying causes of death, foci of infections) are presented in the supplement (Table S1).

### Relevant predictors for mortality

**Fig 1:**
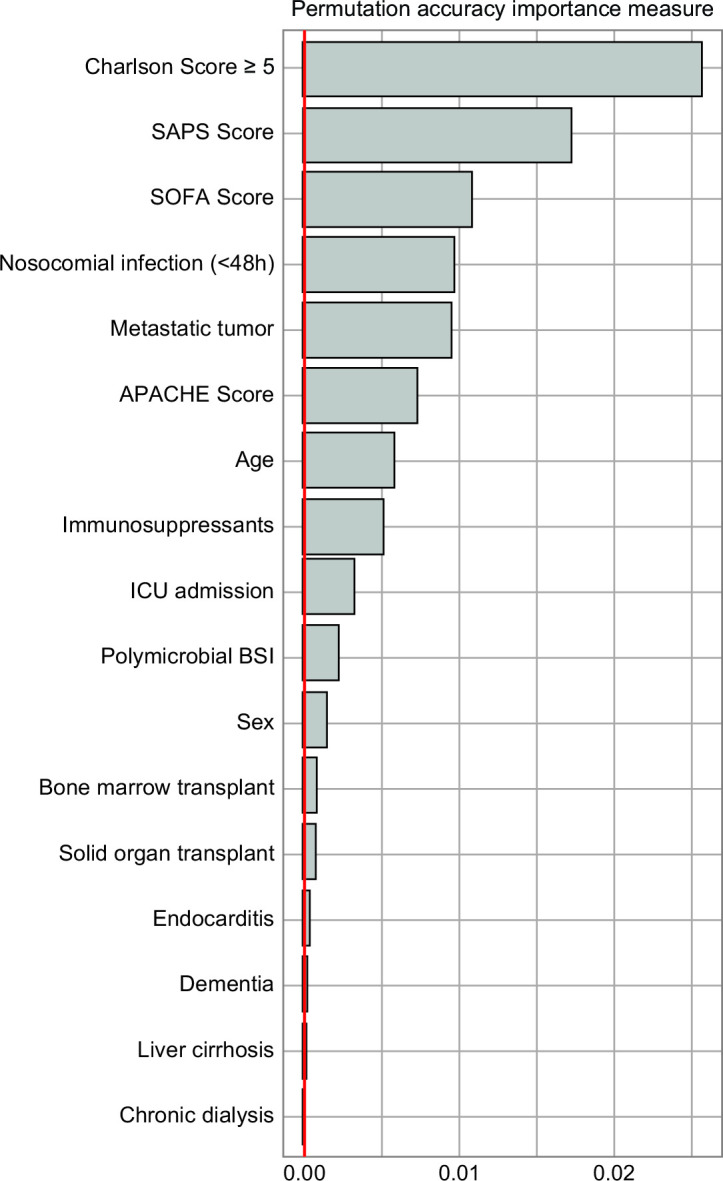
The most relevant prognostic factors for mortality in patients with enterococcal BSI identified by a random forest model (the red line represents the zero point).

The importance of prognostic factors for the prediction of mortality in enterococcal BSI was assessed using a RFM (Fig. 1). The extent of comorbidities (CCI ≥5) and markers for severity of Illness [Acute Physiology and Chronic Health Evaluation (APACHE)-II score, Sequential Organ Failure Assessment (SOFA) score, and Simplified Acute Physiology Score (SAPS)-II)] were the most relevant factors associated with mortality, followed by nosocomial BSI, age, immunosuppressive therapy, and sex.

### Survival outcome (Cox proportional hazards model)

Overall, patients with non-recurrent enterococcal BSI had a significantly increased overall mortality compared to those with *E. coli* BSI (hazard ratio: 1.66; 95% confidence interval: 1.35–2.04, *P* < 0.001).

**Fig 2:**
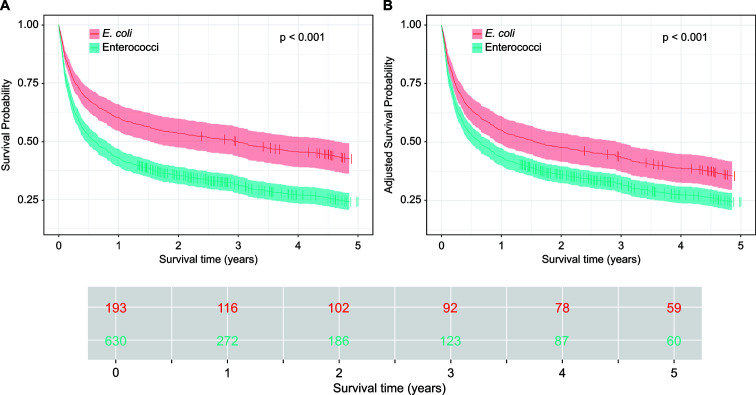
Survival probability of *E. coli* BSI and enterococcal BSI over a 5-year follow-up period calculated by a Cox proportional hazards regression model. (A) unadjusted analysis. (B) Analysis adjusted to 17 prognostic factors* for mortality as illustrated in the RFM.

The 5-year survival (median follow-up: 25 months; range: 1–134 months) after BSI were 42.3% for non-recurrent *E. coli* BSI (35.1% for non-ESBL *E*. coli) and 23.9% for non-recurrent enterococcal BSI (*P* < 0.001, Fig. 2). This difference remained significant even after adjusting for 17 additional risk factors for mortality (*E. coli*: 35.4%, enterococci: 24.4%; *P* < 0.001) and in subgroups after excluding patients with polymicrobial BSI (*E. coli*: 42.8%, enterococci: 23.5%; *P* < 0.001) as well as those with limited life expectancy, such as patients with CCI ≥5 and/or metastatic tumor disease (*E. coli*: 44.1%, enterococci: 27.0%; *P* < 0.001).

Patients with recurrent enterococcal BSI had a 5-year survival of 32.4%.

**Fig 3:**
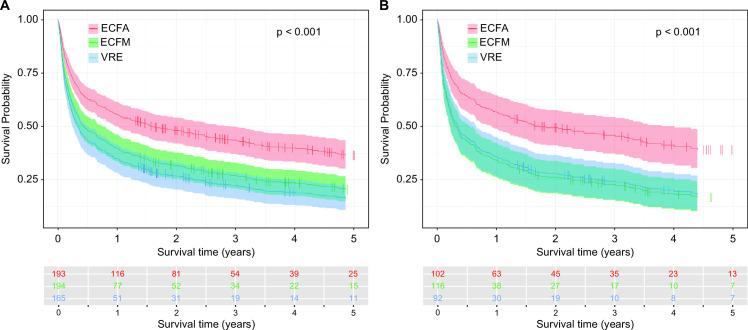
(A) Unadjusted analysis by a Cox proportional hazards regression model (*P* < 0.001) illustrating long-term survival on enterococcal subgroup level over a 5-year follow-up period. (Not shown: Adjusted analysis to 17 prognostic factors* for mortality as illustrated in the RFM: *P* = 0.002). (**B**) Unadjusted analysis by a Cox proportional hazards regression model excluding polymicrobial BSI (*P* < 0.001) showing long-term survival on enterococcal subgroup level over a 5-year follow-up period. (Not shown: Adjusted analysis to 17 prognostic factors* for mortality as illustrated in the RFM: *P* = 0.004).

At the *Enterococcus* subgroup level, the 5-year survival of patients with non-recurrent BSI was 36.4% (adjusted: 29.2%) for ECFA, 20.4% (adjusted: 21.7%) for ECFM, and 16.2% (adjusted: 18.2%) for VRE (Fig. 3A, *P* < 0.001, adjusted *P* = 0.002). The exclusion of patients with polymicrobial episodes from the analysis revealed the highest 5-year survival for ECFA (38.9%, adjusted: 30.9%), followed by VRE (18.1%, adjusted: 22.3%) and ECFM (16.7%, adjusted: 18.0%) (Fig. 3B, *P* < 0.001, adjusted *P* = 0.004).

**Fig 4:**
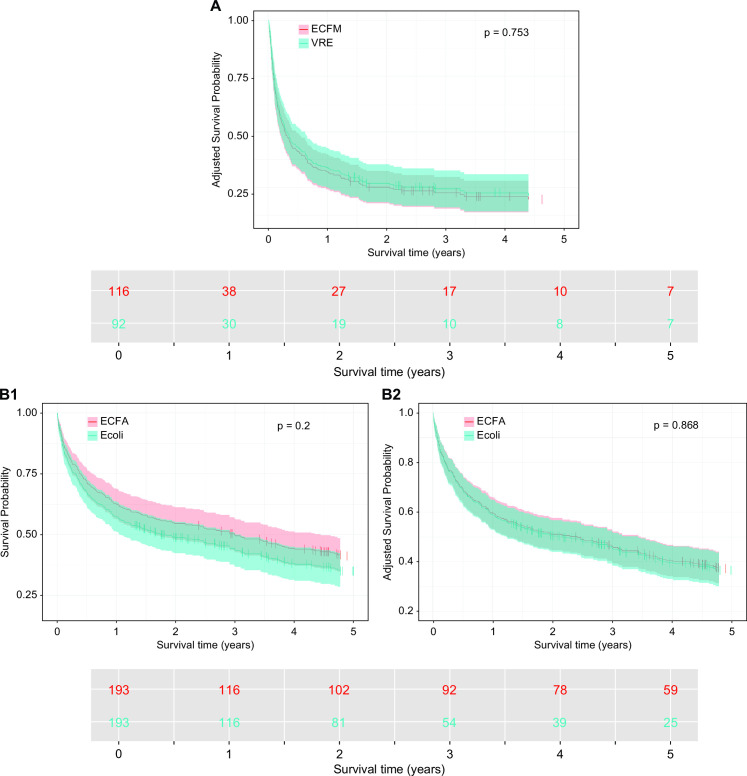
(A) Analysis by Cox proportional hazards regression models comparing 5-year long-term survival between ECFM BSI and VRE BSI (polymicrobial BSI excluded). (Not shown: Adjusted analysis to 17 prognostic factors* for mortality as illustrated in the RFM: *P* = 0.371). (**B**) Analysis by a Cox proportional hazards regression model illustrating the difference in 5-year long-term survival between ECFA BSI and *E. coli* BSI. B1: Unadjusted analysis. B2: Analysis adjusted to the 17 contributing factors* for mortality as illustrated by RFM. *CCI, SAPS-II, SOFA score, nosocomial BSI, metastatic tumor diseases, APACHE-II score, age, immunosuppressants, admission to the ICU, polymicrobial BSI, sex, bone marrow transplantation, solid organ transplantation, endocarditis, dementia, liver cirrhosis, and chronic hemodialysis. Abbreviations: ECFM: *Enterococcus faecium*; ECFA: *Enterococcus faecalis*; VRE: vancomycin-resistant Enterococcus.

Cox proportional hazards regression model analysis within subgroups restricted to non-recurrent ECFM and VRE BSI revealed no significant difference in 5-year survival, both in analyses including polymicrobial BSI (*ECFM* 19.6%, VRE 21.2%; *P* = 0.321, adjusted *P* = 0.300) and after excluding polymicrobial BSI, as shown in Fig. 4A (ECFM 19.6%, VRE 21.2%; *P* = 0.753, adjusted *P* = 0.371).

The 5-year survival (Fig. 4B) for patients with non-recurrent *E. coli* and ECFA BSI was 35.1% (adjusted: 36.4%) and 41.3% (adjusted: 37.2%), respectively, with no significant difference between the two groups (*P* = 0.2, adjusted: *P* = 0.868). An analysis in subgroups that excluded BSI with ESBL-producing *E. coli* revealed consistent findings, with a 5-year survival of 35.1% (adjusted: 35.9%) for *E. coli* and 42.9% (adjusted: 38.6%) for ECFA, with no significant difference (*P* = 0.1, adjusted *P* = 0.568).

The 90-day short-term survival is illustrated in the supplement.

## DISCUSSION

Previous studies suggested that the high mortality of enterococcal BSI, particularly in the presence of multi-resistant enterococci, is attributed to the underlying disease severity or the extent of comorbidities rather than to the pathogen itself (18 – 20). This retrospective multicenter study analyzed the long-term outcomes of enterococcal BSI, taking into account the respective enterococcal species as well as the antimicrobial resistance pattern, and compared them with the outcomes of *E. coli* BSI. As expected, measures for assessing the extent of comorbidities (CCI) and disease severity (APACHE-II score, SOFA score, and SAPS-II) were the most relevant factors contributing to mortality for enterococcal BSI when analyzed by RFM.

Concerning non-recurrent BSI, a significantly lower survival for enterococcal BSI than for *E. coli* BSI was observed. This significant difference was still present after adjustment for 17 additional risk factors illustrated by the RFM and excluding polymicrobial episodes as well as patients with a limited life expectancy (CCI ≥5, metastatic tumor disease). This finding suggests that enterococcal BSI are an independent risk factor for death compared to other bacterial BSI. The lower 5-year long-term survival in enterococcal BSI might be related to the extent of dysbiosis in the gut microbiome, which is thought to be associated with poorer long-term outcomes (21 – 23). It has been shown that the microbiota of patients changes during hospitalization and after antibiotic exposure leading to a predominance of *Clostridiaceae*, *Enterococcaceae,* and *Enterobacteriaceae* (24). Moreover, in patients who underwent stem cell transplantation (SCT), intestinal dominance by VRE occurred before VRE BSI (23). Increased mortality was also observed in patients undergoing SCT who experienced a loss of microbiota diversity, with dominance by single taxa, particularly the genera *Enterococcus* and *Streptococcus*.

At the E*nterococcus* subgroup level, multiple Cox proportional hazards regression model analysis revealed a significantly lower 5-year survival for non-recurrent ECFM than for ECFA. The 5-year survival of *E. coli* BSI was almost identical to that of ECFA BSI. No significant difference in the 5-year survival was observed between patients with ECFM and those with VRE. Thus, based on our findings, we could not show that vancomycin resistance increased long-term mortality in ECFM BSI. Similar findings were observed by Kramer et al. (16), who analyzed the effect of vancomycin resistance on survival in a multicenter cohort study with over 1,000 enterococcal BSI. In contrast to patients with ECFA, the 5-year long-term survival of patients with ECFM BSI was significantly lower. However, their study did not find any differences in survival between vancomycin-resistant and vancomycin-susceptible ECFM BSI. They concluded that the in-hospital mortality due to enterococcal BSI might rather be attributed to the underlying *Enterococcus* species than to vancomycin resistance. Interestingly, this difference in long-term survival cannot be observed in other common, clinically relevant, Gram-positive pathogens with similar resistance patterns. Methicillin-resistant *Staphylococcus aureus* (MRSA) and ECFM share a similar resistance pattern, as β-lactam antibiotics are ineffective against both pathogens. An observational cohort study by Yaw et al. (25) on the long-term survival of MRSA and methicillin-susceptible *S. aureus* BSI found no difference in survival after adjusting for comorbidities and illness severity.

The retrospective nature of the current study is a limitation. Furthermore, the data collection period from 2010 to 2019 was quite long, which was mainly related to the low incidence of multidrug-resistant ECFM (VRE, LVRE, and LRE) BSI in Munich. However, the large number of uncomplicated enterococcal (ECFA, vancomycin/linezolid-susceptible ECFM) and *E. coli* BSI during this period made consecutive inclusion of this subgroup impractical. To minimize selection bias, patients with ECFA- and vancomycin/linezolid-susceptible ECFM BSI were randomly included. Considering that enterococcal BSI tend to occur mostly in severely ill patients with multiple medical conditions (19, 26), *E. coli* BSI were randomly selected with a predefined composition of 40% ICU patients, 30% hemato-oncology patients, and 30% general ward patients to guarantee a similar level of severity of illness. Thus, from the present study, it cannot be concluded whether patients with enterococcal BSI are, in general, more severely ill or have more comorbidities than those with *E. coli* BSI. Additionally, because of the limited number of LVRE or LRE BSI, we could not perform a reliable Cox proportional hazards regression analysis to compare long-term outcomes between patients with ECFM and those with LRE/LVRE infections.

In summary, the main objective of this study was to investigate whether there are any differences in long-term survival between patients with *E. coli* and enterococcal BSI and whether the underlying antimicrobial resistance pattern and species have any impact on the long-term survival in patients with enterococcal BSI. Our study demonstrated that even after adjusting for disease severity, underlying comorbidities, and considering other factors, such as polymicrobial or recurrent BSI, the 5-year long-term survival for patients with enterococcal BSI was lower than that for patients with *E. coli* BSI. This difference in 5-year long-term survival was primarily due to the infections caused by ECFM, whereas ECFA BSI had long-term outcomes similar to those for *E coli* BSI. Furthermore, we did not observe a significant difference in the 5-year long-term survival between patients with ECFM and those with VRE BSI.

## MATERIALS AND METHODS

### Study design

This retrospective multicenter study included adult patients (aged ≥18 years) with enterococcal BSI from seven hospitals in Munich, in Germany (Munich Municipal Hospital Group, University Hospital rechts der Isar of the Technical University of Munich, and University Hospital of the Ludwig Maximilian University of Munich) between 2010 and 2019. The hospitals have a combined total of more than 6,000 patient beds and offer tertiary medical care in Munich, Germany.

All patients with complicated enterococcal BSI were included in the analysis. Complicated enterococcal BSI was defined as any of the following:

1. positive blood culture (BC) with LRE, VRE, and LVRE
2. BSI with two different enterococcal species within one episode
3. recurrent enterococcal BSI with more than one BSI episode within 30 days

**Fig 5:**
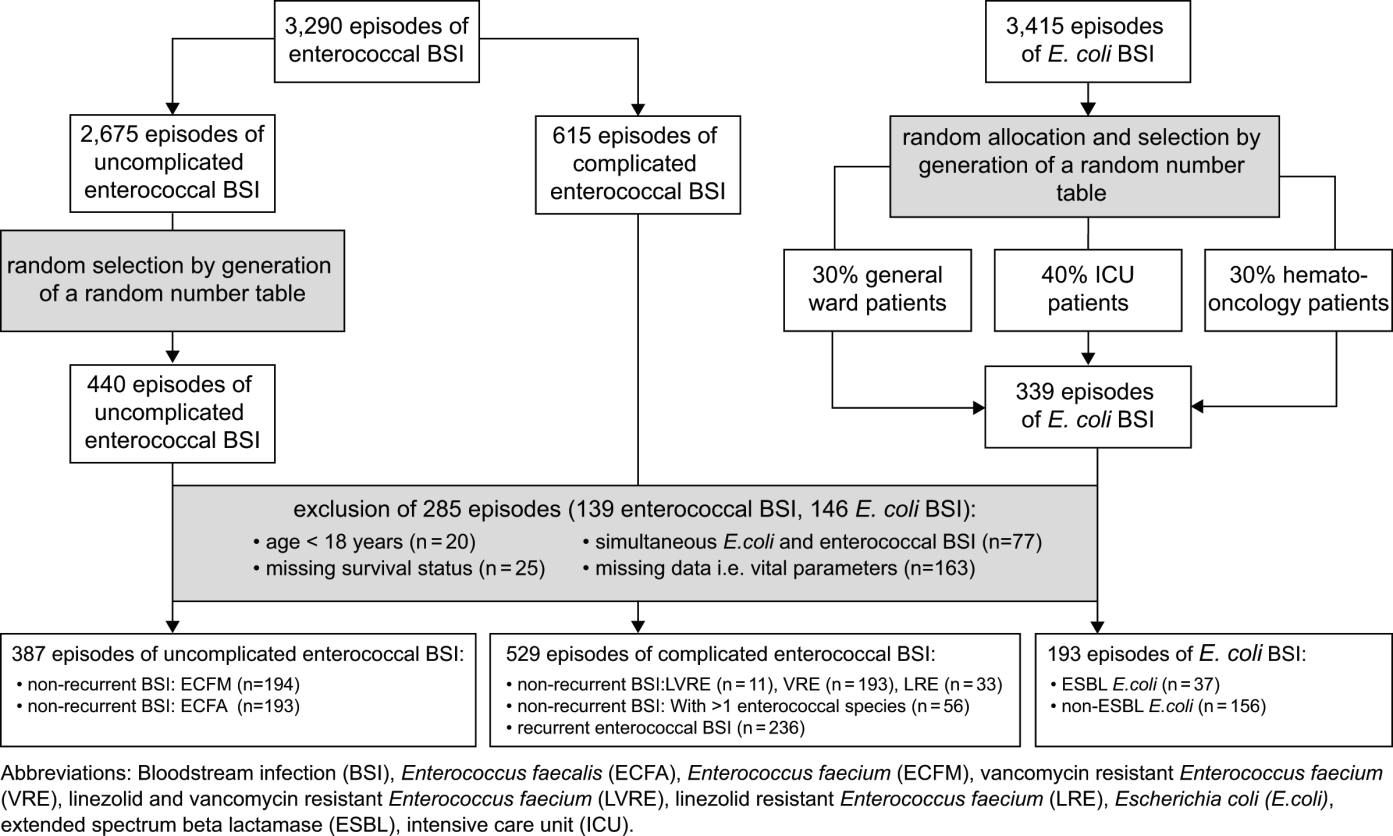
The flow chart depicting patient selection.

Patients were considered to have a separate enterococcal BSI episode if they had another positive BC for *Enterococcus* species ≥30 days after the initial positive BC. Uncomplicated enterococcal BSI were defined as BSI caused by ECFA or ECFM strains that were susceptible to both vancomycin and linezolid and did not meet the abovementioned criteria for complicated enterococcal BSI. The number of uncomplicated enterococcal BSI was large (random sample *n* = 2,675). Therefore, to ensure a representative sample size, patients with uncomplicated enterococcal BSI were selected using a random number table generated in R (version 4.0.3; R Foundation for Statistical Computing, Vienna, Austria). Patients with *E. coli* BSI hospitalized during the same observation period served as the control group. Additionally, patients with *E. coli* BSI were also randomly allocated to constitute 40% intensive care unit (ICU) patients, 30% hemato-oncological patients, and 30% general ward patients to achieve an equivalent level of illness severity compared to patients with enterococcal BSI. Out of 3,290 enterococcal and 3,415 *E. coli* BSI episodes, 1,055 enterococcal and 339 *E. coli* BSI episodes were included, and 285 BSI episodes were excluded. The major reasons for exclusion were unknown survival status due to place of residence outside of the Federal German State of Bavaria (*n* = 25), age <18 years (*n* = 20), coincidence of *E. coli* BSI and enterococcal BSI (*n* = 77), and missing clinical data, such as vital parameters (*n* = 163), which were required for Acute Physiology and Chronic Health Disease Classification System II (APACHE-II) Scoring. Fig. 5 illustrates the patient recruitment process in detail.

### Data collection

Data extraction was performed using the HyBase analysis software (epiNet AG, Bochum, Germany) as well as the electronic medical records and laboratory information management systems. Demographic and clinical data included age, demographic characteristics, presence of comorbidities (chronic cardiac failure, chronic pulmonary and renal diseases, diabetes, hematologic malignancies, inflammatory disease, and solid cancer), Charlson Comorbidity Index, Sequential Organ Failure Assessment (SOFA) score, APACHE-II score, clinical parameters, community-acquired vs nosocomial BSI, prior known enterococcal colonization, use of antibiotics during the previous 3 months, hospitalization (within the previous 3 months), source of infection, presence of central venous catheters at the time of BSI, type of ward, laboratory findings, microbiological analysis (BC results and antimicrobial susceptibilities), inpatient management, details on hospital stay as well as antibiotic regimen and (long-term) mortality including the cause of death. Nosocomial BSI were defined as BSI occurring ≥48 hours after hospital admission. Information on microbiological diagnostics is provided in the supplements.

### Follow-up

Follow-up was started for all patients on the first day of BSI. The final day of follow-up for all outcome measures and deaths was 20 April 2021. The central death registry database of the Munich public health department was used to assess case fatalities. All death certificates in the death registry database and post-mortem reports were assessed for the underlying cause of death.

### Statistical analysis

The distribution of continuous variables is described using medians and ranges, and categorical data are presented as absolute and relative frequencies. Chi-square and *t*-tests were used for group comparisons of categorical and continuous variables, respectively. A random forest based on the “MissForest” algorithm was used to impute missing values (27). The supplements contain information on sample size calculation.

The outcome measure was survival time. For the analysis, we considered the first episode of enterococcal or *E. coli* BSI as the index episode of bacteremia. In cases of two different enterococcal species within one episode, the most resistant *Enterococcus* subtype was used to define the subgroup. To rank the relevance of clinical predictors for mortality in enterococcal BSI, permutation accuracy importance measures were calculated using a Random Forest prediction model (RFM)(28, 29). A multiple Cox proportional hazards regression model was fit to assess the 5-year long-term survival, with- and without-effect estimates adjusted for the following prognostic risk factors: CCI, Simplified Acute Physiology Score II , SOFA score, nosocomial BSI, metastatic tumor diseases, APACHE-II score, age, immunosuppressants, admission to the ICU, polymicrobial BSI, sex, bone marrow transplantation, solid organ transplantation, endocarditis, dementia, liver cirrhosis, and chronic hemodialysis. All variables were dichotomized, except for SAPS-II, SOFA score, and APACHE-II score. The cut-off for the CCI was set at ≥5 points. Analyses were performed at the enterococcal species level. To account for confounding factors, confounder-adjusted survival curves were constructed using the Cox proportional hazards model (30). The causes of death in patients with enterococcal and *E. coli* BSI are presented as the relative frequency of occurrence. In contrast to our Cox proportional hazards regression models, these frequencies do not account for censoring and competing risks, are only descriptive, and should not be confused with risks.

Exploratory statistical hypothesis testing was performed using two-sided significance levels of α = 0.05. All analyses and web applications were implemented using R version 4.0.3.

## Data Availability

Raw data were generated at the Technical University of Munich, School of Medicine, Munich, Germany, and are available from the corresponding author upon request.

## SUPPLEMENTAL MATERIAL

Supplemental data spectrum.02585-23-s0001.docx Supplemental text and Table S1.

Graphical abstract spectrum.02585-23-s0002.eps Supplemental graphic.

ASM does not own the copyrights to Supplemental Material that may be linked to, or accessed through, an article. The authors have granted ASM a non-exclusive, world-wide license to publish the Supplemental Material files. Please contact the corresponding author directly for reuse.
