# Erratum for Rothe et al., “Are enterococcal bloodstream infections an independent risk factor for a poorer 5-year survival or just a marker for severity of illness?—The Munich multicentric enterococci cohort”

**DOI:** 10.1128/spectrum.00951-24

**Published:** 2024-05-07

**Authors:** Kathrin Rothe, Tobias Bachfischer, Siranush Karapetyan, Alexander Hapfelmeier, Milena Wurst, Sabine Gleich, Karl Dichtl, Roland M. Schmid, Julian Triebelhorn, Laura Wagner, Johanna Erber, Florian Voit, Rainer Burgkart, Andreas Obermeier, Ulrich Seybold, Dirk H. Busch, Patrick C. Rämer, Christoph D. Spinner, Jochen Schneider

## ERRATUM

Volume 11, no. 6, e02585-23, 2023, https://doi.org/10.1128/spectrum.02585-23. Page 1, byline: Ulrich Seybold’s surname should appear as shown in this erratum.

Page 10, Author Contributions: “Seibold” should read “Seybold.”

