# Supplementary material for: Are enterococcal bloodstream infections an independent risk factor for a poorer 5-year survival or just a marker for severity of illness?—The Munich multicentric enterococci cohort
Source: Microbiol Spectr. 2023 Oct 4;11(6):e02585-23. doi: 10.1128/spectrum.02585-23 (PMC10715215; doi:10.1128/spectrum.02585-23)
Supplement: Supplemental data — Supplemental text and Table S1. [file spectrum.02585-23-s0001.docx]

**Supplements**

**Methods**

**Microbiological analysis**

Blood culture (BC) samples were processed using aerobic and anaerobic media (BACTEC™ Plus, Becton Dickinson, Sparks, MD, USA) via an automated BC system. The culture bottles were incubated for 5−7 days following the manufacturer's instructions. The identification of pathogens and susceptibility testing were performed using gram staining, MALDI-TOF (Matrix Assisted Laser Desorption Ionisation-Time of Flight Mass Spectrometry, Bruker Daltronics, Leipzig, Germany), automated antimicrobial susceptibility testing (VITEK®, bioMerieux, Marcy l'Étoile, France), minimal inhibitory concentration test strips (Liofilchem Inc., Waltham, MA, USA), and polymerase chain reaction for suspected Vancomycin-resistant *Enterococci* (GeneXpert, Cepheid, CA, USA).

**Sample size**

Review of the literature (1–6) revealed a mean 30-day mortality for enterococcal and *E. coli* BSI to be 25.6% and 13.8%, respectively. The 30-day mortality rate among *Enterococcus* species ranged from 57% for VRE and 34% for ECFM to 21% for ECFA(6–9). Assuming these differences in mortality over a period of 5 years, a sample size of at least 139 patients with *E. coli* BSI and 378 patients with enterococcal BSI (ECFA: n=139, ECFM: n=139, VRE: n=139) would be necessary to achieve a power of 90% for a large sample test of proportions using a two-sided significance level of 5% (10).

**Results**

**Baseline characteristics (univariate analysis)**

A total of 65.0% of the included patients were male, with a median age of 65 years. Patients with enterococcal BSI were treated in the ICU more frequently (58.0% vs. 48.7%, p=0.012). The mean Charlson Comorbidity Index (CCI) was higher in the enterococcal BSI group (5.073 vs. 4.223, p<0.001), while the APACHE-II score, SOFA score, and SAPS-II were similar between the groups. Patients with enterococcal BSI more frequently had healthcare-associated/nosocomial BSI (67.5% vs. 57.5%, p=0.007) and had been hospitalised more often within the previous 3 months before BSI (69.0% vs. 56.6%, p<0.001) compared to those with *E. coli* BSI. The most common foci of infection in patients with enterococcal BSI were abdominal (50.5%), urogenital (21.8%), and foreign body-associated (19.3%). Regarding *E. coli* BSI, the most frequent foci of infection were febrile neutropenia (27.5%) and urogenital infections (26.9%), followed by abdominal infections (25.9%). The underlying cause of infection was un-identified in 25 episodes of *E. coli* (13.0%) and 149 episodes of enterococcal BSI (16.3%). Overall mortality was higher in the enterococcal BSI group compared to patients with *E. coli* BSI (71.7% vs. 60.6%, p=0.003). The overall mortality was higher in ECFM BSI (overall: 75.3% VRE: 77.2%; LVRE: 81.8%; LRE: 97.0%) than in ECFA BSI (62.2%). All baseline characteristics are presented in Table S1.

**Short-term (90-day) Survival outcome (Cox proportional hazards model)**

Short-term (90-day) survival was 76.7% for *E. coli* BSI (76.7% for non-ESBL *E. coli*) and 64.4% for enterococcal BSI(p<0.001). The 90-day survival for VRE, ECFM, and ECFA BSI were 56.2%, 60.4%, and 72.6%, respectively (p<0.001). After the exclusion of polymicrobial episodes, the 90-day survival of patients was 77.7% for *E. coli BSI* (76.9% for non-ESBL) and 62.7% for *enterococcal BSI* (p<0.001), with 55.3% for VRE, 53.8% for ECFM, and 72.1% for *ECFA* (p<0.001).

**Underlying causes of death**

During a median follow-up of 25 (range 1−134) months, 117/193 patients (60.6%) with *E. coli* BSI and 657/916 patients (71.7%) with enterococcal BSI died. The underlying cause of death could not be conclusively determined because of missing documentation in 9.3% (61/657) of patients with enterococcal BSI and 28.2% (33/117) of those with *E. coli* BSI. Analysis of death certificates and post-mortem reports revealed that deaths from infectious causes among patients with enterococcal- and *E. coli-* BSIs, were 329/657 (50.1%) and 44/117 (37.6%), respectively. Non-infectious causes of death were also noted, with the number of patients (n=134) dying due to malignant disease being almost twice as high in the enterococcal BSI group than in the *E. coli* BSI group (20.4% vs. 12.8%). The incidences of cardiac-related deaths (5.8% vs. 5.1%) and deaths after haemorrhagic shock (3.0% vs. 1.7%) were similar between both BSI groups. The incidence of cerebral causes of death was higher in patients with *E. coli* BSI than in those with enterococcal BSI (7.7% vs. 2.7%). Of the patients with *E. coli* and enterococcal BSI, 6.9% (8/117) and 8.7% (57/657), respectively, had other causes of death, such as organ failure or not otherwise specified, multiple traumata, intoxication, or suicide.

**Table S1:** Baseline characteristics of enterococcal and *E. coli* BSI, including univariate analysis

| Baseline characteristics  [n/N (%)] unless otherwise indicated | Total Cohort | Patients with *E. coli*  BSI | Patients with enterococcal  BSI | p-value | Patients with ECFM BSI | Patients with VRE* BSI | p-value | Patients with ECFA* BSI | p-value | Patients with LVRE* BSI | p-value | Patients with LRE* BSI | p-value |
| --- | --- | --- | --- | --- | --- | --- | --- | --- | --- | --- | --- | --- | --- |
| Episodes | 1109 | 193 (17.4) | 916  (82.6) | - | 194 | 193 | - | 193 | - | 11 | - | 33 | - |
| Patients | 952 | 193 (20.3) | 759 (79.7) | - | 194 | 187 | --- | 193 | - | 11 | - | 33 | - |
| Male Sex | 721 (65.0) | 113 (58.5) | 608 (66.4) | 0.046 | 116 (59.8) | 123 (63.7) | 0.740 | 143 (74.1) | 0.004 | 9  (81.8) | 0.346 | 23 (69.7) | 0.587 |
| Age  [Median (25^th^ and 75^th^ percentile)] | 68.00 (56.00; 76.00) | 66.00 (57.00; 74.00) | 68.00 (56.00; 76.00) | 0.901 | 67.00 (57.25; 76.00) | 68.00 (59.00; 75.00) | 0.356 | 70.00 (59.00; 79.00) | 0.005 | 68.00 (63.50; 72.50) | 0.531 | 58.00 (53.00; 69.00) | 0.021 |
| Mortality | 774 (69.8) | 117 (60.6) | 657 (71.7) | 0.003 | 146 (75.3) | 149 (77.2) | 0.016 | 120 (62.2) | 0.012 | 9  (81.8) | 0.520 | 32 (97.0) | 0.001 |

- Comparison of group characteristics between ECFM and other enterococcal species/resistance phenotypes

| Scores  [n/N (%)] unless otherwise indicated | Total Cohort | Patients with *E. coli*  BSI | Patients with enterococcal  BSI | p-value | Patients with ECFM BSI | Patients with VRE* BSI | p-value | Patients with ECFA* BSI | p-value | Patients with LVRE* BSI | p-value | Patients with LRE* BSI | p-value |
| --- | --- | --- | --- | --- | --- | --- | --- | --- | --- | --- | --- | --- | --- |
| APACHE-II score  [Median (25^th^ and 75^th^ percentile)] | 17.00 (13.00; 27.00) | 16.00 (12.25; 30.50) | 17.00 (13.00; 27.00) | 0.728 | 21.00 (13.25; 29.00) | 23.00 (14.00; 28.00) | 0.011 | 15.00 (11.75; 24.00) | 0.002 | 19.50 (17.00; 21.75) | 0.800 | 29.00 (24.00; 30.00) | 0.004 |
| SAPS-II  [Median (25^th^ and 75^th^ percentile)] | 43.00 (34.00; 54.00) | 43.50 (33.00; 58.5) | 42.50 (34.00; 54.00) | 0.513 | 47.50 (40.00; 57.25) | 43.00 (35.00; 52.00) | 0.602 | 35.00 (30.00; 42.00) | <0.001 | 53.00 (50.00; 58.00) | 0.265 | 50.00 (39.00; 66.00) | 0.080 |
| SOFA score  [Median (25^th^ and 75^th^ percentile)] | 4.00 (2.00; 8.00) | 4.00 (2.00; 6.00) | 4.00 (2.00; 9.00) | 0.691 | 4.00 (2.00; 12.00) | 4.50 (2.00; 11.00) | 0.007 | 3.00 (1.00; 7.00) | 0.025 | 4.00 (2.00; 7.50) | 0.885 | 9.00 (5.50; 15.00) | 0.009 |
| CCI  [Median (25^th^ and 75^th^ percentile)] | 4.00 (3.00; 6.00) | 4.00 (3.00; 5.00) | 5.00 (3.00; 7.00) | <0.001 | 5.00 (3.00; 7.00) | 5.00 (3.00; 7.00) | 0.235 | 5.00 (3.00; 7.00) | 0.561 | 4.00 (3.00; 5.00) | 0.064 | 4.00 (2.00; 5.00) | 0.385 |

| Comorbidities/  Diseases  [n/N (%)] unless otherwise indicated | Total Cohort | Patients with *E. coli*  BSI | Patients with enterococcal  BSI | p-value | Patients with ECFM BSI | Patients with VRE* BSI | p-value | Patients with ECFA* BSI | p-value | Patients with LVRE* BSI | p-value | Patients with LRE* BSI | p-value |
| --- | --- | --- | --- | --- | --- | --- | --- | --- | --- | --- | --- | --- | --- |
| Endocarditis | 47 (4.2) | 1 (0.5) | 46 (5.0) | 0.005 | 3 (1.5) | 7 (3.6) | 0.701 | 12 (6.2) | 0.166 | 0 (0) | 1.000 | 1 (3.0) | 1.000 |
| Dementia | 44 (4.0) | 7 (3.6) | 37 (4.0) | 0.844 | 8 (4.1) | 10 (5.2) | 0.415 | 14 (7.3) | 0.015 | 0 (0) | 1.000 | 0 (0) | 0.397 |
| Chronic dialysis | 52 (4.7) | 2 (1.0) | 50 (5.5) | 0.007 | 10 (5.2) | 14 (7.3) | 0.089 | 10 (5.2) | 0.852 | 1 (9.1) | 1.000 | 3 (9.1) | 0.398 |
| Bone marrow transplantation | 83  (7.5) | 31 (16.1) | 52  (6.4) | <0.001 | 16  (8.2) | 21 (10.9) | 0.257 | 5  (2.6) | 0.001 | 1  (9.1) | 1.000 | 5  (15.2) | 0.195 |
| Solid organ transplantation | 95  (8.6) | 7  (3.6) | 88  (9.6) | 0.007 | 12  (6.2) | 23 (11.9) | 0.088 | 9  (4.7) | 0.033 | 1  (9.1) | 1.000 | 4  (12.1) | 0.520 |
| Active tumour disease | 525 (47.3) | 84 (43.5) | 441 (48.1) | 0.267 | 107 (55.2) | 107 (55.4) | 0.428 | 83 (43.0) | 0.003 | 7  (63.6) | 0.553 | 18 (54.5) | 0.861 |
| Metastatic tumour disease | 136 (12.3) | 11 (5.7) | 125 (13.6) | 0.002 | 32 (16.5) | 20 (10.4) | 0.401 | 27 (14.0) | 0.468 | 0  (0) | 0.378 | 4  (12.1) | 1.000 |
| Liver cirrhosis | 70 (6.3) | 15 (7.8) | 55 (6.0) | 0.414 | 18 (9.3) | 11 (5.7) | 0.749 | 5 (2.6) | 0.021 | 0 (0) | 0.636 | 2 (6.1) | 1.000 |
| Medical immunosuppression | 232 (20.9) | 29 (15.0) | 203 (22.2) | 0.032 | 34 (17.5) | 57 (29.5) | 0.001 | 25 (13.0) | 0.003 | 3  (27.3) | 0.708 | 14 (42.4) | 0.004 |
| CCI >5 | 547 (49.3) | 73 (37.8) | 474 (51.7) | <0.001 | 108 (55.7) | 104 (53.9) | 0.178 | 100 (51.8) | 0.476 | 3  (27.3) | 0.225 | 12 (36.4) | 0.158 |

| Type of BSI / admission  [n/N (%)] unless otherwise indicated | Total Cohort | Patients with *E. coli*  BSI | Patients with enterococcal  BSI | p-value | Patients with ECFM BSI | Patients with VRE* BSI | p-value | Patients with ECFA* BSI | p-value | Patients with LVRE* BSI | p-value | Patients with LRE* BSI | p-value |
| --- | --- | --- | --- | --- | --- | --- | --- | --- | --- | --- | --- | --- | --- |
| Nosocomial bloodstream infection | 729 (65.7) | 111 (57.5) | 618 (67.5) | 0.010 | 155 (79.9) | 165 (85.5) | <0.001 | 100 (51.8) | <0.001 | 9  (81.8) | 0.349 | 29 (87.9) | 0.008 |
| Prior hospitalisation within 3 months | 666 (60.1) | 109 (56.6) | 557 (69.0) | <0.001 | 147 (75.8) | 147 (76.2) | 0.021 | 110 (57.0) | <0.001 | 11 (100) | 0.042 | 23 (69.7) | 1.000 |
| Length of hospital stay (d) [Median (25^th^ and 75^th^ percentile)] | 29.00 (14.00; 62.00) | 22.00 (10.00; 38.00) | 31.00 (15.00; 69.00) | <0.001 | 32.00 (19.00; 60.00) | 40.00 (21.00; 73.00) | 0.023 | 20.00 (9.00; 39.00) | <0.001 | 66.00 (36.00; 92.50) | 0.191 | 73.00 (30.00; 100.00) | 0.024 |
| ICU admission | 626 (56.2) | 94 (48.7) | 531 (58.0) | 0.021 | 123 (63.4) | 136 (70.5) | <0.001 | 96 (49.7) | 0.046 | 9  (81.8) | 0.126 | 26 (78.8) | 0.011 |
| Mechanical ventilation | 411 (37.1) | 66 (34.2) | 345 (42.8) | 0.054 | 98 (50.5) | 103 (53.4) | 0.001 | 74 (38.3) | 0.262 | 7  (63.6) | 0.218 | 20 (60.6) | 0.032 |
| Resuscitation | 72 (6.5) | 10 (5.2) | 62 (7.7) | 0.285 | 17 (8.8) | 19 (9.8) | 0.120 | 8 (4.1) | 0.090 | 2 (18.2) | 0.178 | 3 (9.1) | 0.724 |

| Microbiological parameters  [n/N (%)] unless otherwise indicated | Total Cohort | Patients with *E. coli*  BSI | Patients with enterococcal  BSI | p-value | Patients with ECFM BSI | Patients with VRE* BSI | p-value | Patients with ECFA* BSI | p-value | Patients with LVRE* BSI | p-value | Patients with LRE* BSI | p-value |
| --- | --- | --- | --- | --- | --- | --- | --- | --- | --- | --- | --- | --- | --- |
| Polymicrobial BSI | 444 (40.1) | 38 (19.7) | 407 (44.4) | <0.001 | 78 (40.2) | 88 (45.6) | 0.090 | 91 (47.2) | 0.029 | 7  (63.6) | 0.129 | 17 (51.5) | 0.207 |
| ESBL-producing *E. coli* |  | 37 (19.2) |  |  |  |  |  |  |  |  |  |  |  |
| *Klebsiella spp.* | 100 (9.0) | 8 (4.1) | 92 (10.0) | 0.012 | 10 (5.2) | 22 (11.4) | 0.213 | 20 (10.4) | 0.489 | 1  (9.1) | 1.000 | 3  (9.1) | 1.000 |
| *Citrobacter spp.* | 15 (1.4) | 1 (0.5) | 14 (1.5) | 0.337 | 1 (0.5) | 4 (2.1) | 0.490 | 3 (1.6) | 1.000 | 0 (0) | 1.000 | 0 (0) | 1.000 |
| *Enterobacter spp.* | 79 (7.1) | 8 (4.1) | 71 (7.8) | 0.090 | 8 (4.1) | 17 (8.8) | 0.354 | 18 (9.3) | 0.217 | 0 (0) | 0.624 | 3 (9.1) | 0.726 |
| *Proteus spp.* | 27 (2.4) | 6 (3.1) | 21 (2.3) | 0.606 | 3 (1.5) | 1 (0.5) | 0.069 | 10 (5.2) | 0.011 | 0 (0) | 1.000 | 0 (0) | 0.628 |
| *Serratia spp.* | 9 (0.8) | 1 (0.5) | 8 (0.9) | 0.713 | 1 (0.5) | 2 (1.0) | 1.000 | 0 (0) | 0.232 | 0 (0) | 1.000 | 0 (0) | 1.000 |
| *Pseudomonas spp.* | 34 (3.1) | 2 (1.0) | 32 (3.5) | 0.103 | 4 (2.1) | 8 (4.1) | 0.357 | 6 (3.1) | 1.000 | 1 (9.1) | 0.291 | 2 (6.1) | 0.621 |
| *Acinetobacter spp.* | 3 (0.3) | 0 (0) | 3 (0.3) | 0.643 | 0 (0) | 0 (0) | 0.643 | 2 (1.0) | 0.080 | 0 (0) | 1.000 | 0 (0) | 1.000 |
| *Bacteroides spp.* | 9 (0.8) | 1 (0.5) | 8 (0.9) | 0.713 | 1 (0.5) | 1 (0.5) | 0.713 | 3 (1.6) | 0.373 | 0 (0) | 1.000 | 0 (0) | 1.000 |
| *Staphylococcus aureus* | 39 (3.5) | 5 (2.6) | 34 (3.7) | 0.526 | 11 (5.7) | 9 (4.7) | 0.387 | 5 (2.6) | 0.526 | 0 (0) | 1.000 | 3 (9.1) | 0.106 |
| Coagulase negative *Staphylococci* | 84 (7.6) | 8 (4.1) | 76 (8.3) | 0.051 | 18 (9.3) | 20 (10.4) | 0.133 | 16 (8.3) | 0.765 | 0 (0) | 0.619 | 4 (12.1) | 0.307 |
| Viridans group *Streptococci* | 29 (2.6) | 6 (3.1) | 23 (2.5) | 0.805 | 5 (2.6) | 2 (1.0) | 0.145 | 7 (3.6) | 0.456 | 0 (0) | 1.000 | 0 (0) | 0.624 |
| *Candida albicans* | 53 (4.8) | 2 (1.0) | 51 (5.6) | 0.008 | 16 (8.2) | 13 (6.7) | 0.191 | 2 (1.0) | 0.008 | 3 (27.3) | 0.013 | 2 (6.1) | 1.000 |
| *Candida spp.* other than *C. albicans* | 50 (4.5) | 2 (1.0) | 48 (5.2) | 0.011 | 10 (5.2) | 15 (7.8) | 0.022 | 3 (1.6) | 0.033 | 1 (9.1) | 0.399 | 6 (18.2) | 0.003 |
| Other yeasts | 3 (0.3) | 0 (0) | 3 (0.3) | 0.643 | 1 (0.5) | 0 (0) | 0.643 | 0 (0) | 0.643 | 0 (0) | 1.000 | 0 (0) | 1.000 |
| Other Anaerobes | 12 (1.1) | 2 (1.0) | 10 (1.1) | 1.000 | 2 (0.1) | 2 (1.0) | 1.000 | 3 (1.6) | 0.703 | 0 (0) | 1.000 | 0 (0) | 1.000 |
| Other Gram-positives | 26 (2.3) | 3 (1.6) | 23 (2.5) | 0.461 | 4 (2.1) | 7 (3.6) | 0.292 | 4 (2.1) | 0.808 | 1 (9.1) | 0.231 | 1 (3.0) | 1.000 |
| Non ECFA/ECFM *Enterococci* | 15 (1.4) | 2 (1.0) | 13 (1.4) | 0.756 | 2 (0.1) | 3 (1.6) | 1.000 | 3 (1.6) | 1.000 | 1 (9.1) | 0.140 | 0 (0) | 1.000 |
| Other *Streptococci* | 4 (0.4) | 0 (0) | 4 (0.4) | 0.607 | 2 (0.1) | 0 (0) | 0.607 | 1 (0.5) | 1.000 | 0 (0) | 1.000 | 0 (0) | 1.000 |
| Nonfermenting Gram-negatives | 16 (1.4) | 1 (0.5) | 15 (1.6) | 0.332 | 2 (0.1) | 1 (0.5) | 0.332 | 1 (0.5) | 0.332 | 1 (9.1) | 0.148 | 1 (3.0) | 0.385 |

- Comparison of group characteristics between ECFM and other enterococcal species/resistance phenotypes

Abbreciations: BSI: bloodstream infection; VRE: vancomycin-resistant Enterococcus; ECFM: *E. faecium;* ECFA: *E. faecalis;* LRE: linezolid-resistant *E. faecium;* LVRE: vancomycin/linezolid-resistant *E. faecium*

**References**

1. Tocut M, Zohar I, Schwartz O, Yossepowitch O, Maor Y. 2022. Short- and long-term mortality in patients with urosepsis caused by Escherichia coli susceptible and resistant to 3rd generation cephalosporins. BMC Infect Dis 22**:**571.

2. Richelsen R, Smit J, Schonheyder HC, Laxsen Anru P, Gutiérrez-Gutiérrez B, Rodríguez-Bãno J, Nielsen H. 2020. Outcome of community-onset ESBL-producing Escherichia coli and Klebsiella pneumoniae bacteraemia and urinary tract infection: a population-based cohort study in Denmark. J Antimicrob Chemother 75:3656–3664.

3. Abernethy JK, Johnson AP, Guy R, Hinton N, Sheridan EA, Hope RJ. 2015. Thirty day all-cause mortality in patients with Escherichia coli bacteraemia in England. Clin Microbiol Infect 21:251 e1–8.

4. Suppli M, Aabenhus R, Harboe ZB, Andersen LP, Tvede M, Jensen JU. 2011. Mortality in enterococcal bloodstream infections increases with inappropriate antimicrobial therapy. Clin Microbiol Infect 17:1078–1083.

5. Billington EO, Phang SH, Gregson DB, Pitout JD, Ross T, Church DL, Laupland KB, Parkins MD. 2014. Incidence, risk factors, and outcomes for Enterococcus spp. blood stream infections: a population-based study. Int J Infect Dis 26:76–82.

6. Pinholt M, Ostergaard C, Arpi M, Bruun NE, Schønheyder HC, Gradel KO, Søgaard M, Knudsen JD; Danish Collaborative Bacteraemia Network (DACOBAN). 2014. Incidence, clinical characteristics and 30-day mortality of enterococcal bacteraemia in Denmark 2006-2009: a population-based cohort study. Clin Microbiol Infect 20:145–151.

7. Xie O, Slavin MA, Teh BW, Bajel A, Douglas AP, Worth LJ. 2020. Epidemiology, treatment and outcomes of bloodstream infection due to vancomycin-resistant enterococci in cancer patients in a vanB endemic setting. BMC Infect Dis 20:228.

8. Hemapanpairoa J, Changpradub D, Thunyaharn S, Santimaleeworagun W. 2021. Does vancomycin resistance increase mortality? Clinical outcomes and predictive factors for mortality in patients with *Enterococcus faecium* infections. Antibiotics (Basel) 10:105.

9. Edmond MB, Ober JF, Dawson JD, Weinbaum DL, Wenzel RP. 1996. Vancomycin-resistant enterococcal bacteremia: natural history and attributable mortality. Clin Infect Dis 23:1234–1239.

10. Chow S, Shao J, Wang, H. 2008. Sample size calculations in clinical research. 2nd ed. Chapman & Hall/CRC Biostatistics Series.

1. Tocut M, Zohar I, Schwartz O, Yossepowitch O, Maor Y. Short- and long-term mortality in patients with urosepsis caused by Escherichia coli susceptible and resistant to 3rd generation cephalosporins. *BMC Infect Dis* 2022; **22**(1): 571.

2. Richelsen R, Smit J, Schonheyder HC, et al. Outcome of community-onset ESBL-producing Escherichia coli and Klebsiella pneumoniae bacteraemia and urinary tract infection: a population-based cohort study in Denmark. *J Antimicrob Chemother* 2020; **75**(12): 3656-64.

3. Abernethy JK, Johnson AP, Guy R, Hinton N, Sheridan EA, Hope RJ. Thirty day all-cause mortality in patients with Escherichia coli bacteraemia in England. *Clin Microbiol Infect* 2015; **21**(3): 251 e1-8.

4. Suppli M, Aabenhus R, Harboe ZB, Andersen LP, Tvede M, Jensen JU. Mortality in enterococcal bloodstream infections increases with inappropriate antimicrobial therapy. *Clin Microbiol Infect* 2011; **17**(7): 1078-83.

5. Billington EO, Phang SH, Gregson DB, et al. Incidence, risk factors, and outcomes for Enterococcus spp. blood stream infections: a population-based study. *Int J Infect Dis* 2014; **26**: 76-82.

6. Pinholt M, Ostergaard C, Arpi M, et al. Incidence, clinical characteristics and 30-day mortality of enterococcal bacteraemia in Denmark 2006-2009: a population-based cohort study. *Clin Microbiol Infect* 2014; **20**(2): 145-51.

7. Xie O, Slavin MA, Teh BW, Bajel A, Douglas AP, Worth LJ. Epidemiology, treatment and outcomes of bloodstream infection due to vancomycin-resistant enterococci in cancer patients in a vanB endemic setting. *BMC Infect Dis* 2020; **20**(1): 228.

8. Hemapanpairoa J, Changpradub D, Thunyaharn S, Santimaleeworagun W. Does Vancomycin Resistance Increase Mortality? Clinical Outcomes and Predictive Factors for Mortality in Patients with Enterococcus faecium Infections. *Antibiotics (Basel)* 2021; **10**(2).

9. Edmond MB, Ober JF, Dawson JD, Weinbaum DL, Wenzel RP. Vancomycin-resistant enterococcal bacteremia: natural history and attributable mortality. *Clin Infect Dis* 1996; **23**(6): 1234-9.

10. Chow S, Shao, J. and Wang, H. Sample Size Calculations in Clinical Research. 2nd Ed. Chapman & Hall/CRC Biostatistics Series. . 2008.
